# Evaluation of the Effectiveness of Clinical Pharmacists’ Consultation in the Treatment of Infectious Diseases: A Single-Arm, Prospective Cohort Study

**DOI:** 10.3389/fphar.2019.00187

**Published:** 2019-03-01

**Authors:** Jiaxing Zhang, Xin Qian, Lingmin Zhang, Linfang Hu, Lingyan Fan, Qingchen Wang, Bo Lan, Changcheng Sheng, Li Li, Wenyi Zheng, Juan Xie

**Affiliations:** ^1^Department of Pharmacy, Guizhou Provincial People’s Hospital, Guiyang, China; ^2^Experimental Cancer Medicine, Clinical Research Center, Department of Laboratory Medicine, Karolinska Institute, Stockholm, Sweden

**Keywords:** infectious diseases, clinical pharmacists’ consultation, cohort study, registry database, service

## Abstract

**Background:** With the implementation of Antimicrobial Stewardship Program, clinical pharmacists’ consultation (CPC) for infectious diseases (ID) is gradually adopted by many hospitals in China. We conducted a cohort study to evaluate the effectiveness of CPC in ID treatment on patient outcomes and potential determinants.

**Methods:** Based on a registry database, a prospective cohort study was conducted in Guizhou Provincial People’s Hospital. The main exposure factor was whether clinician adopted the suggestion from clinical pharmacist. The outcome was effective response rate (ERR) of ID patients. The variables associated with the outcome (e.g., age, gender, severity of infection, liver function, and kidney function) were also prospectively recorded. A multilevel model was performed to analyze the factors related to ERR.

**Results:** A total of 733 ID inpatients were included in the final analysis according to the predesigned inclusion and exclusion criteria. The proportion of clinical pharmacists’ suggestions adopted by clinicians and ERR were 88.13 and 69.03%, respectively. Significant data aggregation (*P* < 0.05) for individuals at the level of department was observed. According to the two-level variance component model, liver dysfunction (*Adjusted Odds Ratio (AOR)* = 0.649, 95%*Credible Interval* (*CI)*: 0.432–0.976), severity of infection (*AOR* = 0.602, 95%*CI*: 0.464–0.781), and adopting the suggestion from pharmacist (*AOR* = 1.738, 95%*CI*: 1.028–2.940) had significant association with ERR.

**Conclusion:** Our study suggests that the effect of CPC on ID treatment is significant. The policy/decision makers or hospital managers should be cognizant of the critical value of clinical pharmacists in ID treatment.

## Introduction

The severity of infectious diseases (ID) appears increasing due to the overflow of drug-resistant bacteria and abuse of antibiotics (Meek et al., 2015), and antimicrobial resistance (AMR) has become a serious global health challenge (Marston et al., 2016). About 700,000 people die from resistant infections every year (O’Neill, 2016). A systematic review including 19 observational studies from 11 different countries indicated that *Methicillin-resistant Staphylococcus* aureus (MRSA), *multi-resistant Acinetobacter baumannii* (MRAB), and bacteria producing *extended-spectrum β-lactamase* (ESBL) costed $916.61–62908.00, $4644.00–98575.00, and $2824.14–30093.00 per person, respectively (Ling et al., 2017). As predicted, by 2050 there will be more than 10 million deaths per year (Thakur and Gray, 2019) and a cumulative cost to global economic output of 100 trillion USD due to the rise of AMR (O’Neill, 2016). A study indicated that the increase in AMR burden correlated with a 65% increase in human antimicrobial consumption between 2000 and 2015 in 76 countries (Klein et al., 2018). Low and middle-income countries, particularly those in Sub-Saharan Africa and Asia, are currently the largest consumers of antibiotics worldwide in terms of total tons of antibiotics (Klein et al., 2018) and carry the greatest burden of ID (Laxminarayan and Chaudhury, 2016; Gebretekle et al., 2018). A report from China Antimicrobial Surveillance Network showed that, during 2005–2014, carbapenem-resistant rates of *Klebsiella pneumoniae* and *A. baumannii* had increased from 2.4 to 13.4% and 31 to 66.7%, respectively (Hu et al., 2016). Therefore, there is an urgent need to take action to minimize the emergence of AMR bacteria in developing countries (Laxminarayan et al., 2016).

To combat the AMR threat and optimize antimicrobial use, Antimicrobial Stewardship Program (ASP) has been initialized in many countries (Charani et al., 2019). Although countries are at different stages of implementing ASP, studies conducted in low-, middle- and high-income countries [e.g., South Africa (Brink et al., 2016, 2017), Pakistan (Rehman et al., 2018), Thailand (Apisarnthanarak et al., 2015), Brazil (Magedanz et al., 2012), China (Shen et al., 2011; Zhou et al., 2015; Li et al., 2017), Ireland (Dunn et al., 2011), United States (Waters, 2015; Ellis et al., 2016; Zhang X. et al., 2017; Kulwicki et al., 2018), Japan (Ohashi et al., 2018), England (Wickens et al., 2013), Germany (Grill et al., 2011)] demonstrated the critical role of pharmacists in ASP. The various roles include promoting rational use of antibiotics (Apisarnthanarak et al., 2015; Ellis et al., 2016; Kulwicki et al., 2018), reducing antimicrobial agent consumption, AMR, and costs of hospitalization (Shen et al., 2011; Magedanz et al., 2012; Waters, 2015; Zhou et al., 2015; Brink et al., 2016; Li et al., 2017; Zhang X. et al., 2017; Rehman et al., 2018), shortening duration of antimicrobial treatment and length of hospital stay (Dunn et al., 2011; Grill et al., 2011; Shen et al., 2011; Apisarnthanarak et al., 2015; Ellis et al., 2016; Li et al., 2017), improving life quality and hospital mortality (Li et al., 2017; Ohashi et al., 2018; Rehman et al., 2018). In 2011, health administrative authorities in China introduced pharmacist-driven stewardship into ASP (Ministry of Health of China, 2011), which allowed pharmacists to participate in ID treatment and accelerated the development of clinical pharmacists’ consultation (CPC) for ID.

With the continuous implementation of ASP in China, CPC for ID treatment is gradually adopted by many hospitals and increasing number of studies emerge to assess the value of CPC in ID treatment. We have previously performed a systematic review to evaluate the effectiveness of CPC; however, only a few case series (Zhi-Wen et al., 2015) were available and the methodological quality was poor (e.g., small sample size, selection bias, publication bias, recall bias, and confounding bias) (Zhang X. et al., 2017). Further well-designed studies featuring high-quality evidence are warranted to verify the value of CPC for ID. For this aim, we designed a prospective cohort study basing on registry database, with which we also explored important factors associated with patient outcome.

## Materials and Methods

We followed the STROBE Statement for cohort studies (Supplementary Table S1) to conduct the study.

### Study Design and Setting

This prospective single-center, single-arm cohort study was conducted at the Guizhou Provincial People’s Hospital (a tertiary hospital with 2,500 beds) in Guizhou Province in China. The study was approved by the Ethics Committee of the Guizhou Provincial People’s Hospital (2017066, Supplementary Tables S2, S3) and conducted in accordance with the Declaration of Helsinki.

### Participants

The inpatients with ID diagnosis and receiving CPC services from April to December 2017 were recruited consecutively. Subjects meeting the inclusion criteria were included after signing the written informed consent. Exclusion criteria were applied to the following conditions: (1) Clinicians applied for the CPC after the use of special antibiotics (e.g., vancomycin, carbapenem, tigecycline, etc.). In this case, clinicians only asked pharmacists to approve the use of the special antibiotics in order to meet the requirements of the ASP, therefore pharmacist did not participate in the treatment process of patients; (2) The patient died or discharged from hospital before clinical pharmacists submitted the suggestion; and (3) The patients received multiple rounds of CPC service.

### Consultation Intervention

Consultation intervention generally consisted of four steps: (1) The clinician sent out a consultation request to the Department of Clinical Pharmacy when treating patients with complicated ID; (2) The Department of Clinical Pharmacy assigned one pharmacist qualified with specialist board certifications after 1-year residency training to deal with the consultation; (3) The assigned pharmacist replied with treatment suggestion (determine the initial therapeutic scheme or adjust the present one, including type, dose, and frequency of antibiotics) according to patient’s condition, guideline and/or the best evidence, and the benefits and risks of medications; (4) The clinician made the final decision on therapeutic scheme.

### Exposure Factor and Covariates

The main exposure factor was whether the clinician adopted the suggestion from clinical pharmacist. It was identified by comparing the prescriptions of clinician with the suggestion of pharmacist. The hypothesis was that only if the suggestion of pharmacist was adopted by clinician, the patient could receive the consultation intervention. Acceptance referred to that clinician completely or partially adopted the advice of pharmacist in the treatment. Acceptance rate (AR) was the proportion of consultation suggestions adopted by clinicians to the total consultation requests sent by clinicians. Other covariates (e.g., age, gender, condition of infection, severity of infection, etc.) were also recorded prospectively.

### Outcome

Effective response (complete or partial response) was defined as partial or complete resolution of clinically significant signs/symptoms associated with infection, improvement or resolution of computed tomography (CT) or magnetic resonance imaging (MRI) findings, and on proven or negative culture results. Effective response rate (ERR) was the proportion of patients achieving effective response to total patients. Follow-up time was the 3rd–7th day after the consultation intervention completed.

### Data Collection and Management

A registry database specialized for this study was set up and utilized to record and manage the data of patients. Two investigators independently recorded and crosschecked the data. To reduce the detection bias, the outcome assessor was not the clinical pharmacist providing consultation service.

### Statistics

Statistical analysis was conducted using software SPSS 19.0 and MLwiN. Significance level (α) was set as 0.05.

Statistical description was performed according to the type of data: Mean ± Standard deviation (SD) or median and interquartile range was used to describe quantitative data; rate or constituent ratio was used to describe qualitative data.

Univariate analysis: *t*-test or *z*-test was used for quantitative data which met normal distribution, and rank test was used for quantitative data which did not meet normal distribution; unordered categorical data were analyzed by chi-square test; ranked data were analyzed by rank test.

Multivariate analysis: Logistic regression model was performed to explore significant factors associated with the outcome (effective response). This was achieved by treating the effective response as dependent variable while other factors as independent variables. The value of variable in the multivariate analysis was listed in Supplementary Table S2. If the aggregation of data existed at the department level, multilevel logistic regression model (Yang and Li, 2007) was utilized.

## Results

### Participant Recruitment and Characteristics

**FIGURE 1:**
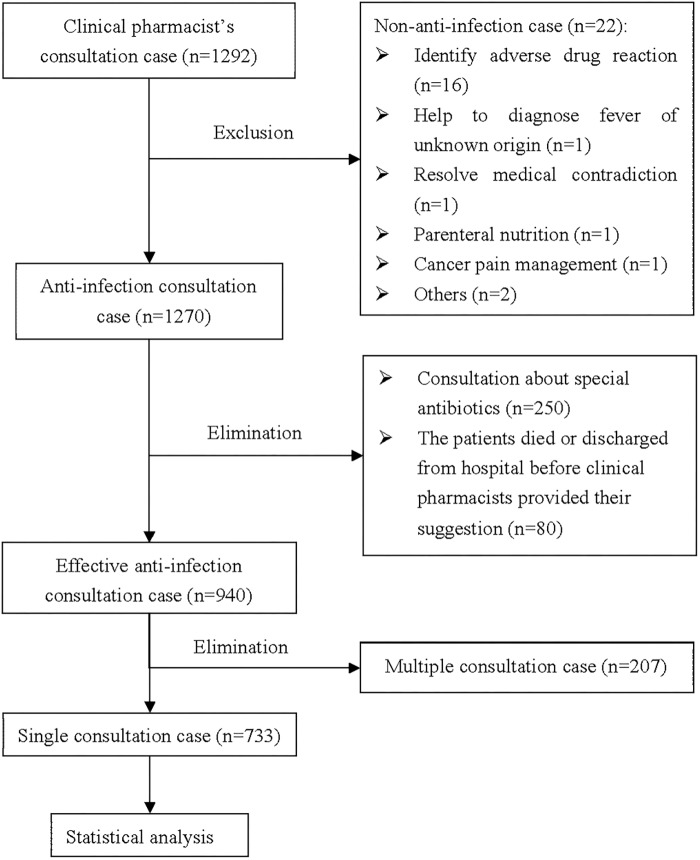
The flow chart of the study.

**FIGURE 2:**
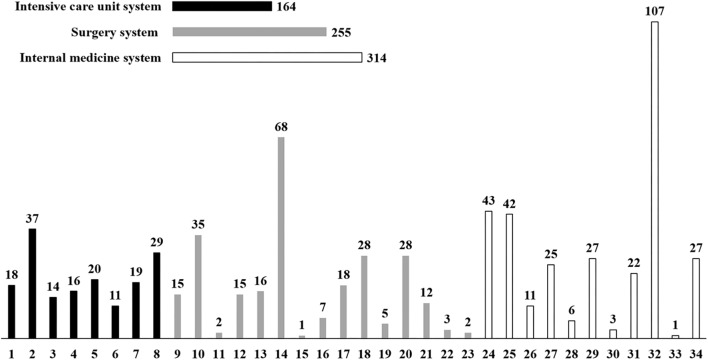
Distribution of included patients among clinical departments. 1: Intracardiac intensive care unit; 2: Emergency intensive care unit; 3: Central intensive care unit; 4: Neurology intensive care unit; 5: Pediatric intensive care unit; 6: Respiratory intensive care unit; 7: Department of Neonatology; 8: Department of Emergency; 9: Department of Obstetrical; 10: Department of Pediatric Surgery; 11: Department of Ear-nose-throat; 12: Department of Gynecology; 13: Department of Hepatobiliary Surgery; 14: Department of Orthopedics; 15: Department of Decorative Surgery; 16: Department of Physiatry; 17: Department of Urinary Surgery; 18: Department of General Surgery; 19: Department of Burn; 20: Department of Neurosurgery; 21: Department of Cardiac Surgery; 22: Department of Thoracic Surgery; 23: Department of Ophthalmology; 24: Department of Pediatrics; 25: Department of Infectious Diseases; 26: Department of Geriatrics; 27: Department of Respiration; 28: Department of Neurology; 29: Department of Nephrology; 30: Department of Gastroenterology; 31: Department of Cardiology; 32: Department of Hematology; 33: Department of Traditional Chinese Medicine; 34: Department of Oncology.

A total of 1,292 patients were screened in the registry database, but only 733 patients meeting the inclusion criteria were included in the final analysis (Figure 1). The baseline characteristics of patients were presented in Figure 2 and Table 1. The patients were from 34 clinical departments, and the highest proportion of patients (14.60%) were from the department of hematology (Figure 2). The number of patients from internal medicine system was higher than surgery system and intensive care unit system.

**Table 1:** Characteristics of patients included in the final analysis (*N* = 733).

Characteristics		*N* (%)
**Male**		441 (60.16)
**Age**		
	*Less than 7 years old*	108 (14.73)
	*7*–*17 years old*	34 (4.64)
	*18*–*40 years old*	131 (17.87)
	*41*–*65 years old*	279 (38.06)
	*More than 65 years old*	181 (24.69)
**With comorbidity**		252 (34.38)
**Hypoalbuminemia**		551 (75.17)
**Abnormal liver function**		174 (23.74)
**Abnormal kidney function**		190 (25.92)
**Time of consultation after hospitalized**		
	<*7 days*	309 (42.16)
	≥*7 days*	424 (57.84)
**Purpose of consultation**		
	*Adjustment of therapeutic regimen*	673 (91.81)
	*Initial therapeutic regimen*	60 (8.19)
**Type of consultation**		
	*Special consultation*	12 (1.64)
	*General consultation*	721 (98.36)
**Major of clinical pharmacist**		
	*Anti-infection*	296 (40.38)
	*Non-anti-infection*	437 (59.62)
**With etiological evidence**		93 (12.69)
**With imaging examination results**		581 (79.26)
**Febrile symptom**		454 (61.94)
**Increased inflammatory indicator**		586 (79.95)
**Hemogram**		
	*Increased*	349 (47.61)
	*Decreased*	120 (16.37)
	*Normal*	264 (36.02)
**Infectious site**		
	*Central nervous system*	20 (2.73)
	*Ear, nose or throat*	6 (0.82)
	*Bloodstream*	42 (5.73)
	*Cardiovascular system*	8 (1.09)
	*Operative incision*	10 (1.36)
	*Skin or soft tissue*	40 (5.46)
	*Urogenital system*	65 (8.87)
	*Bone or joint*	20 (2.73)
	*Intra-abdomen*	88 (12.01)
	*Respiratory system*	370 (50.48)
	*Unclear*	62 (8.46)
**Number of infectious sites**		
	*>1*	175 (23.87)
	*1*	546 (74.49)
	*0*	12 (1.64)
**Surgical treatment of infectious sites**		154 (21.02)
**Type of infection**		
	*Hospital-acquired infection*	251 (34.24)
	*Community-acquired infection*	450 (61.39)
	*Non-infection*	32 (4.37)
**With high risk factors of infection**		449 (61.26)
**Severity of infection**		
	*Serious*	337 (45.98)
	*Moderate*	288 (39.29)
	*Mild*	78 (10.64)
	*None*	30 (4.09)


The proportion of male was 60.16%, and the age of patients was mainly 41 to 65 years old (38.06%). The proportion of patients with abnormal liver function and abnormal kidney function was 23.76 and 25.92%, respectively. 75% of patients suffered from hypoalbuminemia and 34% had other diseases (e.g., cardiac disease, diabetes, hypertension, cancer, chronic obstructive pulmonary disease, coronary atherosclerosis, cerebral infarction, etc.) besides infection.

For the type of consultation, most patients (98.36%) received normal consultation, and only less than 2% of patients received special consultation which needed a multi-disciplinary panel to participate in. As to the purpose of consultation, most patients (91.81%) required clinical pharmacists to adjust the anti-infection therapeutic regimen. A majority of clinical pharmacists (60%) were not specialized in ID. 80% of patients had imaging examination results, but only 12% had specific etiological evidences.

For the infectious condition, 62 and 80% of patients were with fever and increased infectious indicator (i.e., C reactive protein, procalcitonin, or interleukin-6), respectively. Most patients (61.26%) were with high risk factors of infection (e.g., hypoimmunity, long-term steroid exposure, with implants, hyperthermia with chills, etc.) and 45.98% had serious infection.

### Acceptance Rate (AR) and Effective Response Rate (ERR)

The clinician completely and partially adopted the suggestion of clinical pharmacists regards the therapeutic regimen of 576 and 70 patients, respectively, giving a total AR of 88.13%. Among the 733 patients, 506 patients attained effective response and the ERR was 69.03%.

### Factors Associated With Outcome

Univariate analyses were based on the variables listed in Table 1 (e.g., gender, age, type of department, type of consultation, major of clinical pharmacists, liver function, kidney function, number and type of infectious site, temperature, etc.). The results (Supplementary Table S3) showed that type of department, age, temperature, hypoalbuminemia, liver function, high risk factor of infection, surgical treatment of infectious sites, and severity of infection significantly affected patients prognosis (effective response) (*P* < 0.05).

Considering the strong correlation between the severity of infection and number of infectious sites, temperature, hemogram, increased infectious indicator, high risk factor of infection, type of infection, we only input the severity of infection instead of other factors into the multivariate analyses model. The result (Table 2) of logistic regression model showed effective response was associated with type of department, liver function, and severity of infection.

**Table 2:** The results of Logistic regression model (*N* = 733).

Variable	*P value*	*Adjusted Odds Ratios*	*95% Credible Interval*
Gender (male)	0.377	0.858	[0.612, 1.205]
Age	0.195	0.913	[0.795, 1.048]
Type of department (comparator: surgery system) ^a^	0.022	–	–
	(*internal medicine system*) 0.011	0.547	[0.343, 0.871]
	(*intensive care unit system*) 0.013	0.526	[0.316, 0.876]
Type of consultation (special consultation)	0.122	3.469	[0.717, 16.772]
Major of clinical pharmacists (anti-infection)	0.823	1.041	[0.731, 1.484]
Liver dysfunction ^a^	0.020	0.633	[0.430, 0.931]
Kidney dysfunction	0.923	1.019	[0.691, 1.504]
Hypoalbuminemia	0.091	0.700	[0.462, 1.059]
With comorbidity	0.557	0.897	[0.625, 1.288]
Surgical treatment of infectious sites	0.409	1.226	[0.756, 1.988]
Severity of infection ^a^	0.000	0.594	[0.464, 0.760]
Adopting the suggestion from clinical pharmacists	0.059	1.622	[0.981, 2.683]
Constant	0.000	15.106	–


*^*a*^Indicated *P* ≤ 0.05.*

Due to significant aggregation of individual data at the level of department (level 2) (*P* < 0.05), two-level variance component model was used to perform multivariate analyses. As shown in Table 3, liver function, severity of infection, and adopting the suggestion of clinical pharmacists had a significant association with the effective response of infectious patients. Dysfunction of liver (*Adjusted Odds Ratio (AOR)* = 0.649, 95% *Credible Interval (CI)* = [0.432, 0.976]) and increased severity of infection (*AOR* = 0.602, 95%*CI* [0.464, 0.781]) had negative influence on the prognosis of patients, while adopting the suggestion of clinical pharmacists (*AOR* = 1.738, 95%*CI* [1.028, 2.940]) could improve patient outcome.

**Table 3:** The results of two-level variance component model (*N* = 733).

	Variable	Estimated Value	Standard Error	*Adjusted Odd Ratios* [95% C*redible Interval]*
	Constant	2.594	0.534	13.383 [4.699, 38.116]
	Type of department (comparator: surgery system)	-0.561	0.345	0.571 [0.290, 0.122]
	*Internal medicine system*	-0.608	0.367	
	*Intensive care unit system*	-0.077	0.090	0.544 [0.265,1.118]
	Age			0.926 [0.776, 0.105]
	Gender (male)	-0.177	0.180	0.838 [0.589, 1.192]
Fixed Portion	Type of consultation (special consultation)	1.039	0.831	2.826 [0.554, 14.408]
	Major of clinical pharmacist (anti-infection)	0.029	0.190	1.029 [0.709, 1.494]
	Kidney dysfunction	0.023	0.212	1.023 [0.675, 1.550]
	Liver dysfunction ^a^	-0.432	0.208	
	With comorbidity	-0.062	0.192	0.649 [0.432, 0.976]
	Severity of infection ^a^	-0.508	0.133	
	Surgical treatment of infectious sites	0.244	0.258	0.940 [0.645, 1.369]
	Hypoalbuminemia	-0.392	0.221	0.602 [0.464, 0.781]
				1.276 [0.770, 2.116]
				0.676 [0.438, 1.042]
	Adopting the suggestion from clinical pharmacists ^a^	0.553	0.268	1.738 [1.028, 2.940]
Random Portion	Variance of level 2	0.267	0.137	1.306 [0.998, 1.708]
	Scale parameter of level 1	1	0.000	-


*^*a*^Indicated *P* ≤ 0.05.*

## Discussion

At present, there are increasing evidences supporting the role and value of clinical pharmacist in the treatment of chronic renal disease (Salgado et al., 2012; Cooney et al., 2015), hypertension (Santschi et al., 2014), coronary heart disease (Cai et al., 2013), osteoporosis (Elias et al., 2011), diabetes (Santschi et al., 2012), chronic obstructive pulmonary disease (Zhong et al., 2014), and depression (Rubio-Valera et al., 2011). As to ID, consultation service is the main intervention of clinical pharmacist in China. However, our previous systematic review indicated there were only a few low-quality studies (case series) (Zhang X. et al., 2017). Randomized controlled trial (RCT) is the golden standard research design to evaluate the efficacy of intervention; however, our institution requires clinical pharmacists to deal with every consultation application raised by clinicians, it is therefore impossible to randomly allocate patients to consultation group or non-consultation group. Instead, we conducted a prospective cohort study basing on the registry database, and the patients were divided into exposure or control group according to the main exposure factor (adopting the suggestion or not). Comparing to previous case series, this prospective study could avoid the recall bias induced by retrospective studies, reduce the selection bias by enrolling patients consecutively, and control the confounding factors by the multilevel model, which converged on evidence of high quality.

To our best knowledge, this is the first prospective cohort study basing on registry database to evaluate the effectiveness of CPC in ID treatment and investigate the factors associated with patient outcome. We found that some factors (liver function, severity of infection, adopting the suggestion of clinical pharmacist) could significantly influence the prognosis of infectious patients. After controlling the confounding factors by multilevel model, adopting the suggestion of clinical pharmacist was clearly shown to improve patient outcome.

A previous study conducted in Guizhou Province in China reported the AR of 89.33%, which was close to the AR in our study (88.13%) (Zhang et al., 2018b). The high rate indicated that pharmacist-led consultation intervention could be practically incorporated into ASP in China. Nevertheless, the ERR (76%) reported in the former study was higher than ours (69.03%). Likely, this is due to that our institution as the medical center of Guizhou Province receives many referral patients from other hospitals in this region. These patients usually suffer from serious infection, complicated comorbidity, as well as long course of disease, which cause worse prognosis and lower ERR.

The prognosis of infectious patients was associated with many factors, and the individual data had significant aggregation at the level of department. This aggregation was mainly due to the similarity of these characteristics (e.g., the comorbidity of patients and the nursing condition) in the same department. Comparing to the result of logistic regression model, the effect of department type in multilevel model was not significant, probably because the effect of department type was decomposed to the level of department (level 2). According to the results of multilevel model, patients with liver dysfunction had worse outcome (*AOR* = 0.649, 95% *CI* [0.432, 0.976]). Besides the direct influence of liver function on the prognosis of ID patients, liver dysfunction would limit the use of many strong antibiotics (e.g., tigecycline), which makes the anti-infection treatment more difficult. On the other hand, the severity of infection was closely related to the prognosis of infection (*AOR* = 0.602, 95%*CI* [0.464, 0.781]). When other confounding factors were controlled, adopting the suggestion of clinical pharmacist could improve patient prognosis (*AOR* = 1.738, 95%*CI* [1.028, 2.940]), which is consistent with the previous meta-analysis (*OR* = 20.84, 95%*CI* [8.57, 50.69]) (Figure S1) and observational research (*AOR* = 8.94, 95%*CI* [3.99, 20.00]) (Zhang et al., 2018a). Since the meta-analysis did not consider the effect of confounding factors on the outcome, and the observational research only included limited confounding factors, so our conclusion is supposed to have better authenticity.

Interestingly, the effect of clinical pharmacists’ major on the outcome was not significant (*AOR* = 1.029, 95%*CI* [0.709, 1.494]), in line with a previous study (*AOR* = 0.70, 95%*CI* [0.42, 1.17]) (Zhang et al., 2018a). This is somehow attributable to the special consultation method in our institution. Briefly, when the number of consultation applications exceeds the capacity of ID pharmacists, a special consultation method relying on both ID and non-ID pharmacists is adopted. In practical, the non-ID pharmacist underwent a systematic antimicrobial training and then provide consultation under the supervision of ID pharmacist. Result indicates that non-ID pharmacists with appropriate educational background and clinical training are competent for consultation service. This consultation method might be generalized to other developing countries which are struggling with the shortage of pharmacists (Katoue et al., 2014; El Hajj et al., 2016; Salim et al., 2016; Bilal et al., 2017).

The limitations of this study must also be acknowledged as follows: Firstly, since randomization method was not used in the present observational study, it is possible to have selection bias due to the imbalance of baseline between exposure group and control group. In addition, this study only included the patients receiving single consultation intervention but not multiple consultation intervention, due to limited sample size. Furthermore, this was a single center study, which limited the representativeness of patients. A multi-center prospective cohort study is warranted to further evaluate the consultation intervention of clinical pharmacist in ID treatment in the future.

The crucial role of pharmacists in promoting safe and cost-effective use of antimicrobial agents has been acknowledged in the healthcare systems of numerous countries (Dunn et al., 2011; Grill et al., 2011; Shen et al., 2011; Northey et al., 2015; Zhou et al., 2015; Brink et al., 2016; Ellis et al., 2016; Okada et al., 2016), but the value of clinical pharmacist in ID treatment is still underestimated in China. In fact, not every ward of hospital is staffed with enough clinical pharmacists and consultation service is the main mode of clinical pharmacists’ intervention in China as a result of weak willingness to hire clinical pharmacists. Our study not only demonstrates the important value of clinical pharmacist in ID treatment, but also provides powerful evidence for policy/decision makers to extend this intervention mode in China. These successful experiences can also be shared with other developing countries where pharmacists are not actively involved in patient healthcare (Sakeena et al., 2018).

## Conclusion

This observational study suggests that CPC is an effective intervention for ID treatment, although it is originated from ASP in China. The policy/decision makers or hospital managers should be cognizant of the critical value of clinical pharmacists in ID treatment and offer a clinically significant role for clinical pharmacists within healthcare systems.

## Author Contributions

JZ, XQ, LZ, LH, LF, QW, BL, CS, and LL collected the data. JZ, WZ, and JX involved in statistical analysis and drafted the manuscript. JZ, XQ, and JX interpreted the data. All authors conceived and designed the study, performed critical revision of the manuscript for important intellectual content, and approved final version of the manuscript to be published including the authorship list.

## Conflict of Interest Statement

The authors declare that the research was conducted in the absence of any commercial or financial relationships that could be construed as a potential conflict of interest.
