# Supplementary material for: Evaluation of the Effectiveness of Clinical Pharmacists’ Consultation in the Treatment of Infectious Diseases: A Single-Arm, Prospective Cohort Study
Source: Front Pharmacol. 2019 Mar 1;10:187. doi: 10.3389/fphar.2019.00187 (PMC6405418; doi:10.3389/fphar.2019.00187)
Supplement: Supplementary file 1 [file Data_Sheet_1.docx]

Table S1 STROBE Statement—Checklist of items that should be included in reports of ***cohort studies***

|  | Item No | Recommendation | Reported on Page # |
| --- | --- | --- | --- |
| **Title and abstract** | 1 | (*a*) Indicate the study’s design with a commonly used term in the title or the abstract | Page 1 |
|  |  | (*b*) Provide in the abstract an informative and balanced summary of what was done and what was found | Page 1 |
| Introduction | | |  |
| Background/rationale | 2 | Explain the scientific background and rationale for the investigation being reported | Page 4-6 |
| Objectives | 3 | State specific objectives, including any prespecified hypotheses | Page 6 |
| Methods | | |  |
| Study design | 4 | Present key elements of study design early in the paper | Page 7 |
| Setting | 5 | Describe the setting, locations, and relevant dates, including periods of recruitment, exposure, follow-up, and data collection | Page 7 |
| Participants | 6 | (*a*) Give the eligibility criteria, and the sources and methods of selection of participants. Describe methods of follow-up | Page 7 and Page 9 |
|  |  | (*b*) For matched studies, give matching criteria and number of exposed and unexposed |  |
| Variables | 7 | Clearly define all outcomes, exposures, predictors, potential confounders, and effect modifiers. Give diagnostic criteria, if applicable | Page 8-9 |
| Data sources/ measurement | 8* | For each variable of interest, give sources of data and details of methods of assessment (measurement). Describe comparability of assessment methods if there is more than one group | Page 9 |
| Bias | 9 | Describe any efforts to address potential sources of bias | Page 9-10 |
| Study size | 10 | Explain how the study size was arrived at | Not applicable |
| Quantitative variables | 11 | Explain how quantitative variables were handled in the analyses. If applicable, describe which groupings were chosen and why | Not applicable |
| Statistical methods | 12 | (*a*) Describe all statistical methods, including those used to control for confounding | Page 10 |
|  |  | (*b*) Describe any methods used to examine subgroups and interactions | Not applicable |
|  |  | (*c*) Explain how missing data were addressed | Not applicable |
|  |  | (*d*) If applicable, explain how loss to follow-up was addressed | Not applicable |
|  |  | (*e*) Describe any sensitivity analyses | Not applicable |
| Results | | |  |
| Participants | 13* | (a) Report numbers of individuals at each stage of study—eg numbers potentially eligible, examined for eligibility, confirmed eligible, included in the study, completing follow-up, and analysed | Fig 1 and Page 10-11 |
|  |  | (b) Give reasons for non-participation at each stage | Fig 1 |
|  |  | (c) Consider use of a flow diagram | Fig 1 |
| Descriptive data | 14* | (a) Give characteristics of study participants (eg demographic, clinical, social) and information on exposures and potential confounders | Page 11-12  Figure 2 and Table 1 |
|  |  | (b) Indicate number of participants with missing data for each variable of interest | Not applicable |
|  |  | (c) Summarise follow-up time (eg, average and total amount) | Not applicable |
| Outcome data | 15* | Report numbers of outcome events or summary measures over time | Page 12 |
| Main results | 16 | (*a*) Give unadjusted estimates and, if applicable, confounder-adjusted estimates and their precision (eg, 95% confidence interval). Make clear which confounders were adjusted for and why they were included | Page 12-13  Table 2 and Table 3 |
|  |  | (*b*) Report category boundaries when continuous variables were categorized | Not applicable |
|  |  | (*c*) If relevant, consider translating estimates of relative risk into absolute risk for a meaningful time period | Not applicable |
| Other analyses | 17 | Report other analyses done—eg analyses of subgroups and interactions, and sensitivity analyses | Not applicable |
| Discussion | | |  |
| Key results | 18 | Summarise key results with reference to study objectives | Page 14-15 |
| Limitations | 19 | Discuss limitations of the study, taking into account sources of potential bias or imprecision. Discuss both direction and magnitude of any potential bias | Page 16-17 |
| Interpretation | 20 | Give a cautious overall interpretation of results considering objectives, limitations, multiplicity of analyses, results from similar studies, and other relevant evidence | Page 15-16 |
| Generalisability | 21 | Discuss the generalisability (external validity) of the study results | Page 17 |
| Other information | | |  |
| Funding | 22 | Give the source of funding and the role of the funders for the present study and, if applicable, for the original study on which the present article is based | Page 2 |

*Give information separately for exposed and unexposed groups.

**Note:** An Explanation and Elaboration article discusses each checklist item and gives methodological background and published examples of transparent reporting. The STROBE checklist is best used in conjunction with this article (freely available on the Web sites of PLoS Medicine at http://www.plosmedicine.org/, Annals of Internal Medicine at http://www.annals.org/, and Epidemiology at http://www.epidem.com/). Information on the STROBE Initiative is available at http://www.strobe-statement.org.

**Table S2. The value of variable in the multivariate analysis**

| **Variable** | **Value** |
| --- | --- |
| **Gender** | *0=Female*  *1=Male* |
| **Age** | *0=Less than 7 years old*  *1=7-17 years old*  *2=18-40 years old*  *3=41-65 years old*  *4=More than 65 years old* |
| **Type of department** | *0, 0=Surgery system*  *1, 0=Internal medicine system*  *0, 1=Intensive care unit system* |
| **Type of consultation** | *0=General consultation*  *1=Special consultation* |
| **Major of clinical pharmacist** | *0=Non-anti-infection*  *1=Anti-infection* |
| **Liver function** | *0= Normal*  *1= Abnormal* |
| **Kidney function** | *0= Normal*  *1= Abnormal* |
| [**Hypoalbuminemia**](http://www.baidu.com/link?url=Mteuig3w7c5sTKbVoUq93DHuOfPJ4A80W0Ef903-a8-XMTYCYCe_8VetS0evzcNnH3XEXgxi7e0a8OSlICAtjX5mUYzy7B6orwZRFXNMy90lPQD0Moosztau4vnFs5Tn) | *0= No*  *1= Yes* |
| **With comorbidity** | *0= No*  *1= Yes* |
| **Surgical treatment of infectious sites** | *0= No*  *1= Yes* |
| **Severity of infection** | *0= None*  *1= Mild*  *2= Moderate*  *3= Serious* |
| **Adopting the suggestion from clinical pharmacist** | *0= No*  *1= Yes* |

**Table S3. The results of univariate analyses (n=733)**

| **Variable** | **Level** | **Non-response**  **（227）** | **Response**  **（506）** | ***P*** |
| --- | --- | --- | --- | --- |
| **Gender** | *Female* | 83 | 209 | 0.225 |
|  | *Male* | 144 | 297 |  |
| **Age ^a^** | *Less than 7 years old* | 25 | 83 | 0.045 |
|  | *7-17 years old* | 8 | 26 |  |
|  | *18-40 years old* | 52 | 79 |  |
|  | *41-65 years old* | 81 | 198 |  |
|  | *More than 65 years old* | 181 | 120 |  |
| **Type of department ^a^** | *Surgery system* | 47 | 208 | 0.000 |
|  | *Internal medicine system* | 115 | 199 |  |
|  | *Intensive care unit system* | 65 | 99 |  |
| **Type of consultation** | *General consultation* | 225 | 496 | 0.228 |
|  | *Special consultation* | 2 | 10 |  |
| **Major of clinical pharmacist** | *Non-infectious diseases* | 134 | 303 | 0.828 |
|  | *Infectious diseases* | 93 | 203 |  |
| **Liver function ^a^** | *Normal* | 156 | 403 | 0.001 |
|  | *Abnormal* | 71 | 103 |  |
| **Kidney function** | *Normal* | 158 | 386 | 0.056 |
|  | *Abnormal* | 69 | 120 |  |
| **Number of infectious sites** | *0* | 2 | 10 | 0.202 |
|  | *1* | 163 | 383 |  |
|  | *>1* | 62 | 113 |  |
| **Temperature ^a^** | *Normal* | 60 | 219 | 0.000 |
|  | *Fever* | 167 | 287 |  |
| **Hemogram** | *Normal* | 72 | 192 | 0.200 |
|  | *Decreased* | 43 | 77 |  |
|  | *Increased* | 112 | 237 |  |
| [**Hypoalbuminemia**](http://www.baidu.com/link?url=Mteuig3w7c5sTKbVoUq93DHuOfPJ4A80W0Ef903-a8-XMTYCYCe_8VetS0evzcNnH3XEXgxi7e0a8OSlICAtjX5mUYzy7B6orwZRFXNMy90lPQD0Moosztau4vnFs5Tn) **^a^** | *No* | 43 | 139 | 0.013 |
|  | *Yes* | 184 | 367 |  |
| **With comorbidity** | *No* | 140 | 341 | 0.132 |
|  | *Yes* | 87 | 165 |  |
| **Inflammatory indicator** | *Normal* | 39 | 108 | 0.193 |
|  | *Increased* | 188 | 398 |  |
| **With high factors of infection ^a^** | *No* | 61 | 233 | 0.000 |
|  | *Yes* | 166 | 283 |  |
| **Surgical treatment of infectious sites ^a^** | *No* | 193 | 386 | 0.007 |
|  | *Yes* | 34 | 120 |  |
| **Type of infection** | *None* | 5 | 27 | 0.070 |
|  | *Hospital-acquired infection* | 135 | 315 |  |
|  | *Community-acquired infection* | 87 | 164 |  |
| **Severity of infection ^a^** | *None* | 5 | 25 | 0.000 |
|  | *Mild* | 11 | 67 |  |
|  | *Moderate* | 72 | 216 |  |
|  | *Serious* | 139 | 198 |  |
| **Adopting the suggestion from clinical pharmacist** | *No* | 32 | 55 | 0.212 |
|  | *Yes* | 195 | 451 |  |

**^a^ indicated *P*≤0.05.**





**Figure S1. Meta-analysis forest plot of effective response rate of patients in 2 groups.**

**SCP: suggestion of clinical pharmacist**
